# Spatio-temporal variability in transmission risk of human schistosomes and animal trematodes in a seasonally desiccating East African landscape

**DOI:** 10.1098/rspb.2023.1766

**Published:** 2024-01-10

**Authors:** Naima C. Starkloff, Teckla Angelo, Moses P. Mahalila, Jenitha Charles, Safari Kinung'hi, David J. Civitello

**Affiliations:** ^1^ Department of Biology, Emory University, Atlanta, GA 30322, USA; ^2^ National Institute of Medical Research Mwanza Center, Mwanza, Tanzania; ^3^ Nelson Mandela African Institute of Science and Technology, Arusha, Tanzania

**Keywords:** schistosomiasis, *Bulinus*, *Schistosoma haematobium*, ephemerality, aestivation, seasonality

## Abstract

Different populations of hosts and parasites experience distinct seasonality in environmental factors, depending on local-scale biotic and abiotic factors. This can lead to highly heterogeneous disease outcomes across host ranges. Variable seasonality characterizes urogenital schistosomiasis, a neglected tropical disease caused by parasitic trematodes (*Schistosoma haematobium*). Their intermediate hosts are aquatic *Bulinus* snails that are highly adapted to extreme rainfall seasonality, undergoing prolonged dormancy yearly. While *Bulinus* snails have a remarkable capacity for rebounding following dormancy, we investigated the extent to which parasite survival within snails is diminished. We conducted an investigation of seasonal snail schistosome dynamics in 109 ponds of variable ephemerality in Tanzania from August 2021 to July 2022. First, we found that ponds have two synchronized peaks of schistosome infection prevalence and observed cercariae, though of lower magnitude in the fully desiccating than non-desiccating ponds. Second, we evaluated total yearly schistosome prevalence across an ephemerality gradient, finding ponds with intermediate ephemerality to have the highest infection rates. We also investigated dynamics of non-schistosome trematodes, which lacked synonymity with schistosome patterns. We found peak schistosome transmission risk at intermediate pond ephemerality, thus the impacts of anticipated increases in landscape desiccation could result in increases or decreases in transmission risk with global change.

## 1. Introduction

Variability in environmental conditions across multiple spatial scales interacts to create highly heterogenous patterns of disease outcomes across space and time [1]. For example, ambient temperature influences key life-history traits of hosts and their parasites in laboratory settings [2,3]. However, in natural settings, temperature can vary substantially at hourly timescales and across microsites. Local-scale factors such as degree of habitat permanence [4–8], availability of vegetation as micro-habitat and nutrients [9] and availability of winter hardy micro-habitats [10,11] can result in highly spatially heterogeneous host–parasite dynamics. Additionally, these dynamics are affected by seasonality, climatic cycles and global climate change [12–14]. For example, disease transmission is often elevated during warmer periods of the year [12]. Disease occurrence is also typically higher in the rainy season than the dry season due to increased host activity and waterbody connectivity [15] or elevated nutrient runoff [16].

Seasonal changes in host activity and dormancy in response to cyclical climatic conditions can results in yearly peaks and troughs in transmission [12]. However, hosts and parasites in different populations experience distinct seasonal fluctuations in rainfall and temperature depending on local-scale biotic and abiotic factors leading to spatially variable disease outcomes [1,12]. In addition to global changes in temperature and rainfall, human activities such as land use change can have local-scale impacts on microclimate conditions of intermediate hosts [17,18]. Considering the spatio-temporal heterogeneity in host-parasite dynamics at the local-scale, we may expect similar spatial variability in responses of hosts and parasites to global change. Anthropogenic environmental perturbations are expected to directly or indirectly increase the risk of disease incidence globally [1,19], however, the impacts on host and parasite outcomes in regions vulnerable to drought are not extensively examined. Thus, it is imperative to better identify possible parasite outcomes in a desiccating landscape.

Schistosomiasis is a neglected tropical disease caused by parasitic trematodes in the genus *Schistosoma* that infect over 200 million people worldwide in addition to the aquatic snails that act as intermediate hosts [20]. Both free-living stages of *Schistosoma haematobium* (which causes urogenital schistosomiasis) require the presence of water, yet this species occurs in African landscapes with extreme rainfall seasonality [21,22]. Small rain catchment ponds, which are created by villagers in rural areas to increase water availability during dry months for home and agricultural use, are ideal habitat for its drought-adapted intermediate snail host, *Bulinus* species [23]. Rainfall seasonality results in dramatic yearly decreases in the depths and area of these ponds, often leaving ponds dry for many months of the year. Pond ephemerality (the tendency of ponds to dry up annually) is impacted by a plethora of factors, such as pond size, depth, orientation in the landscape and human activities. The intensity of ephemerality can influence host persistence and diversity [5,24–26]. Pond desiccation forces hosts and parasites to use adaptive behaviours for survival [27]; for example, *Bulinus* snails undergo dormancy (known as aestivation) in protected microhabitats within dry or drying ponds and have an incredible capacity for population rebounding with the return of water [7,28]. However, schistosome parasite survival is greatly diminished by aestivation in laboratory studies and is understudied in the field [21]. Considering the physiological challenges of aestivation on parasite survival, we hypothesize that ephemerality acts as a dampener to schistosome transmission risk.

Characterizing schistosome transmission risk with varying ephemerality can provide a blueprint of anticipated risk in an increasingly desiccating landscape. Drought intensification in East Africa is predicted with global change [29], with 10–20 million Tanzanians impacted by drought disasters and the Lake Victoria watershed of northern Tanzania intensifying in water scarcity in the last three decades [30]. To investigate the potential for pond desiccation to deter parasite transmission, we carried out a year-round evaluation (August 2021–July 2022) of snail-parasite dynamics in ponds with varying ephemerality across six districts of the Lake Victoria watershed of northern Tanzania. Water catchment ponds are not well contained or treated for exclusive human use, resulting in utilization and contamination by other host species. It is therefore common for *Bulinus* snails to also be infected by trematodes (typically xiphidiocercaria parasites) that infect other animal species as definitive hosts, such as cattle, poultry and wild animals. Thus, we also quantified transmission risk of these non-schistosome trematodes to assess if seasonal dynamics were similar between the two parasite groups.

We evaluate transmission risk in four ways that are commonly measured in schistosome surveillance studies: (i) snail abundance, (ii) the proportion of snails infected, (iii) *per capita* parasite cercariae release and (iv) the total release of parasitic cercariae observed from sampled snails. We first compare the seasonal dynamics of these four metrics between ponds that desiccate fully for at least one monthly survey in the year and ponds that retain water year-round. However, as ephemerality can be characterized beyond a simple dichotomy of ponds that fully desiccate or not, we also assess if yearly infection prevalence differs with intensity of ephemerality (percentage reduction in pond area in a year). Our study identifies time points and intensities of pond ephemerality that present hotspots of transmission of schistosomes and other animal trematodes.

## 2. Methods

### (a) Sampling sites

**Figure 1.**
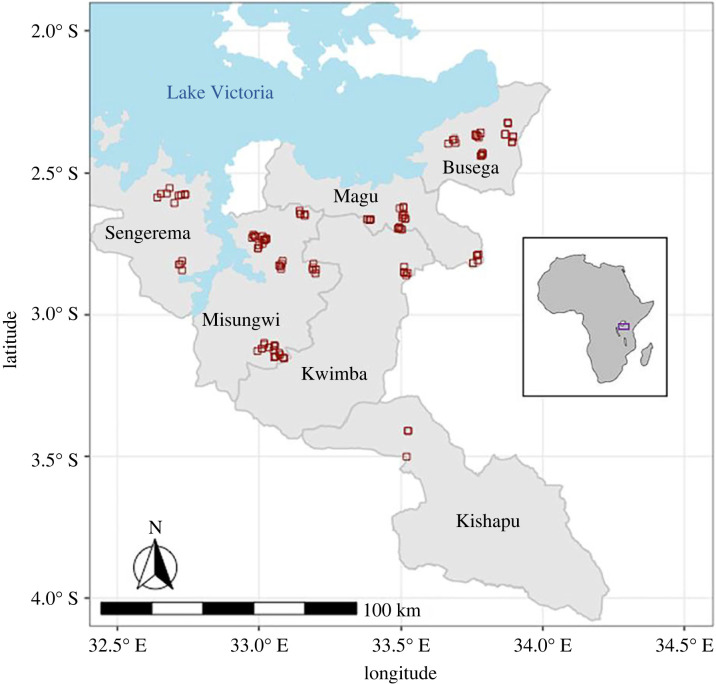
Map of localities of 109 ponds surveyed (red squares) across six districts of northwestern Tanzania, with inset of locality of sites within the continent of Africa.

**Figure 2.**
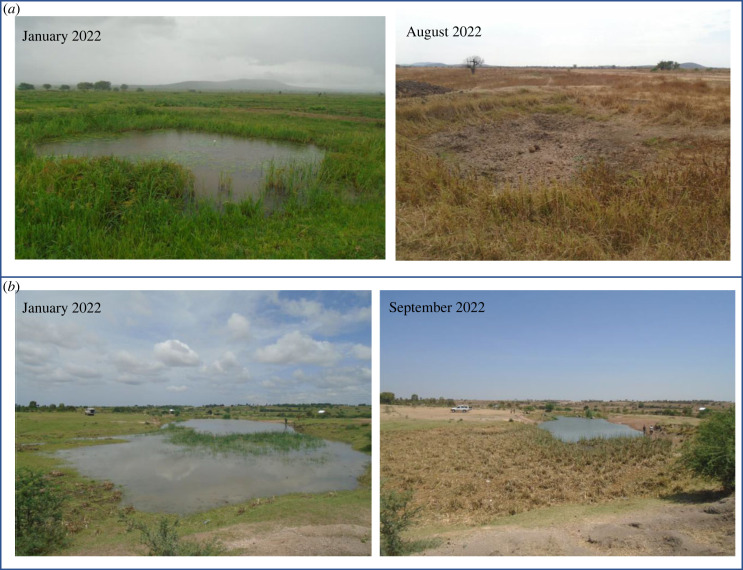
Change in area of (*a*) a desiccating pond in Misungwi district, Kisima cha Longo (reduction of pond area of 100%) and (*b*) a non-desiccating pond in Busega district, Lambo la Wachina (reduction of pond area of 79.78%) within a survey year.

We surveyed 109 ponds monthly in six Tanzanian districts of the Lake Victoria watershed from 23 August 2021 to 27 July 2022 (figure 1). These ponds are created or modified by village communities to increase year-round water availability for the purpose of human household use (Kisima), for cattle use (Lambo) or for longer-term water storage dams with unspecified use (Bwawa). A small number of ephemeral rivers and streams (Mto or Kijito) were also included in the study. Many of these ponds dry completely for several months of the year (figure 2*a*) or dramatically decrease in size (figure 2*b*) in the dry season. All ponds were chosen with approval of, and surveys were conducted in collaboration with, local village leaders. A larger pilot study was conducted in 2020–2021 including 467 pond sites that were identified by local leaders as potential transmission sites across the six districts, based on frequent human and cattle use [31]. In some cases, this included all ponds in the village and in other villages leaders identified six to eight ponds. For the current study, only villages where schistosome-infected snails were found were retained and up to five ponds were retained per village.

**Figure 3.**
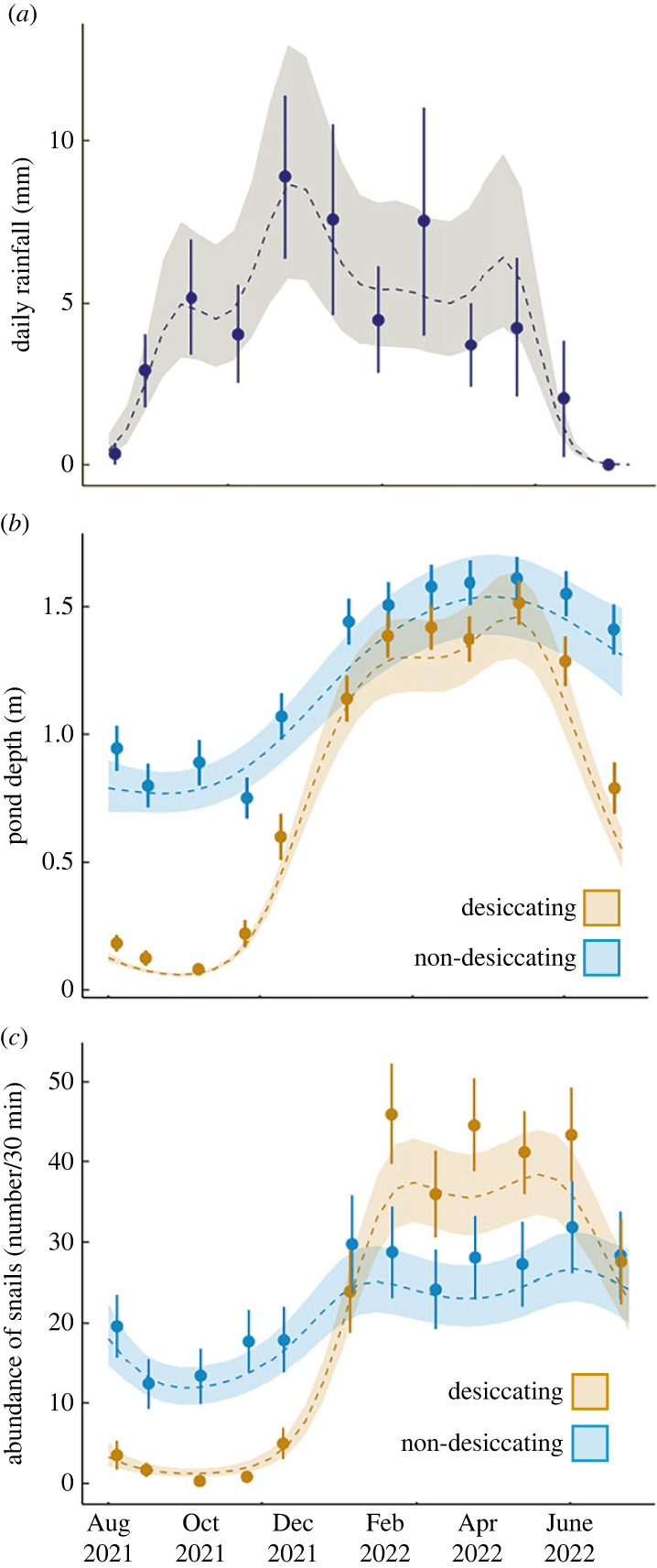
Generalized Additive Models (GAMMs) representing circannual (*a*) rainfall patterns (mm per day) in Mwanza, Tanzania of data from the airport weather station, and variability in (*b*) pond depth (m) and (*c*) *Bulinus nasutus* snail abundance (number collected in 30 min survey) in our 109 sites. The shaded region surrounding the line represents the best fit ± 1 s.e. Points represent monthly means and standard error. Depth and snail abundance were significantly nonlinear and different between the two ephemerality categories.

The Lake Victoria watershed is typically characterized by short and long rainy seasons, *Vuli* (October–December) and *Masika* (March–May), respectively. However, a delayed *Vuli* commencing in December 2021 resulted in a combining of the two rainy periods in our sampling period (figure 3*a*). Rainfall data (in mm per day) was obtained from the Mwanza weather station between 23 August 2021 and 27 July 2022 [32].

During monthly site visits, each pond was surveyed for maximum length and width perpendicular to maximum length in metres using a tape measure, and depth at center in metres using a pole and tape measure. Dry ponds for which these dimensions were 0 metres were noted as such. All ponds that were too deep in the center to measure were assigned a depth of 2 m.

### (b) Snail surveying and collection

We conducted snail surveys in small rainfall catchment areas in northern Tanzania which are occupied by *Bulinus nasutus* snails [22]. This species is morphologically differentiated from other *Bulinus* species in Tanzania, *B. africanus* and *B. globosus*. Two researchers conducted time-constrained net sampling using metal mesh scoop nets to collect *B. nasutus* snails for 15 min (leading to a total of 30 min of surveying per pond per month). This provides a representation of the contact experience of a person standing in the water for 30 min. In addition, this method is as effective as quadrat methods in assessing snail population dynamics across long time periods [33]. Researchers searched for snails across the area of the pond, with special focus on microhabitats (submerged and floating vegetation, and other floating objects). Snails were placed in Nalgene containers in a cooler and brought back to the laboratory at the NIMR Mwanza Centre for cleaning, counting and quantifying parasites (shedding).

### (c) Identifying and quantifying parasite shedding

*Bulinus nasutus* snails were shed for patent infections in individual 30 ml beakers with 25 ml bottled water for 24 h in natural light conditions. Following this full-day shed, beakers were examined under a dissecting microscope at 10–25× for the presence of cercariae (larval forms) of schistosome and non-schistosome trematodes. The cercariae of these two groups are distinguished by size, shape and movement [34]. *Schistosoma haematobium* cannot be morphologically distinguished from *S. bovis* or their hybrids [35], therefore we represented all these individuals as ‘schistosomes’. Non-schistosomes were overwhelmingly represented by xiphiodiocercariae. If the presence of trematodes was confirmed, cercarial intensity was quantified after staining with Lugol's Iodine and homogenization by gentle pipetting. For schistosomes, if the estimated number of cercariae was below 200, all cercariae were counted. If the number was larger, a subsample of 18.5% of the beaker's bottom area was counted and multiplied by 5.412 to extrapolate for the total area of the beaker. For non-schistosome trematodes, only the subsample approach was taken due to higher intensities being typical.

### (d) Identifying potential infected aestivators

We identified if snails carried infections through aestivation using the timing of infections relative to emergence from aestivation in desiccating ponds. The prepatent period that is necessary for infections to develop until cercariae release typically take 6–18 weeks in a laboratory setting for *Schistosoma haematobium* [36]. Ponds need to refill for snails to revive from aestivation and be receptive to miracidia once the water has returned. This could take just a few days or several weeks from the onset of rain, depending on the size of the pond and where in the pond snails are aestivating. As a result, we conservatively infer that infections that were detected less than 60 days following the last dry survey were acquired before aestivation, with increased confidence in those detected less than 30 days after the last dry survey. While *Bulinus* snails aestivate in dry microhabitats within non-desiccating ponds when water conditions are not ideal, it was not possible to identify infected asetivators using our methodology as these ponds did not have any dry surveys as a time reference point.

### (e) Statistical analyses

In this study, we assessed transmission risk in four ways: (i) snail abundance, (ii) the proportion of snails infected, (iii) *per capita* release of parasite cercariae from sampled snails and (iv) the total parasitic cercariae observed from all sampled snails. Snail abundance is defined as the number of *B. nasutus* snails recovered per 30 min pond survey. Thereafter, we assess the proportion of these snails that sheds schistosome or non-schistosome parasites within a 24 h shedding window. In terms of cercarial production, we first looked at the average number of cercariae released by individual snails of each parasite group (*per capita*) and we also summed the total number of cercariae observed of each parasite group by the infected snails that we collected in each pond survey (total observed release).

We used generalized additive mixed models (GAMMs) in the R package mgcv to evaluate how several metrics changed as a function of time and binary pond status (desiccating or non-desiccating). Specifically, we ran individual analyses to test how the following dependent variables changed over the course of a year: water depth (gamma distribution using depth + 0.01 cm and link = ‘log'), snail abundance (Quasi-Poisson distribution) and infection prevalence (binomial distributions) and per capita and total observed cercariae in pond surveys (quasi-Poisson distributions) of the two parasite groups. GAMMs are effective at evaluating smoothed, nonlinear relationships over time. Thus, in each analysis we represented the annual trend as a smooth term. For gamma and binomial error distributions, we fitted models with restricted maximum-likelihood (REML), whereas as for quasi-Poisson models, we fitted with quasi-penalized likelihood [37]. Our independent variable of time is defined as days from the first day of sampling (23 July 2021) ranging from 0 to 338. We fitted models with continuous autoregressive – error structures to account for repeated measures, except for the prevalence and *per capita* cercariae models due to failed convergence. For all models, we fitted the dynamics of non-desiccating waterbodies with a reference temporal smooth and tested for significantly different dynamics in desiccating waterbodies with a temporal difference smooth [37]. Lastly, we included pond ID as a random effect in the GAMMs to account for non-independence in the monthly repeated observations from these replicated sites. All GAMMs were set up with a similar structure to this schistosome prevalence model below. See script for variation per model type.

$$\begin{array}{rl} & >\mathrm{gamm}(\mathrm{cbind}(\mathrm{schisto}\_\mathrm{pos},\;\mathrm{schisto}\_\mathrm{neg})∼s(\mathrm{ContDate})+\\ & s(\mathrm{ContDate},\;\mathrm{by}=\mathrm{Aest})+s(\mathrm{Waterbody},\mathrm{bs}=“\mathrm{re}”),\;\mathrm{family}\\ & =\mathrm{binomial}(“\mathrm{logit}”),\;\mathrm{data}=\mathrm{infected}\_\mathrm{ponds},\;\mathrm{method}=“\mathrm{REML}”) \end{array}$$

We ran generalized linear models (GLMs) with the R package glmmTMB with binomial error distributions on all snails collected from each pond to assess if cumulative yearly infection prevalence varied among the two ephemerality categories. We also ran similar binomial GLMs to assess if yearly transmission risk differs with intensity of ephemerality (% pond reduction in pond area in a year). We summed the total number of infected and uninfected snails per pond per year for each parasite group to evaluate cumulative yearly parasite transmission risk. We then calculated the surface area of each pond on each visit by assuming an elliptical shape (surface area = ½ length × ½ width × π) and the percentage reduction in area of each pond ((max – min) ÷ max × 100) as a measure of ephemerality intensity.

## 3. Results

We collected a total of 30 137 *Bulinus* snails across 12 monthly surveys of 109 ponds, of which 482 snails were infected with schistosome trematodes (1.59%) and 1592 snails were infected with non-schistosome trematodes (5.28%). Ponds were distinguished as desiccating (*n* = 59) or non-desiccating (*n* = 50) depending on whether water was completely absent for at least one monthly survey (figure 2). While non-desiccating ponds had water year-round, water levels contracted between 39.46 and 99.97% the area of these ponds at their lowest observation (figure 2*b*), potentially forcing snails to aestivate due to changes in water temperature, depth and quality [21]. As indicated by the GAMM, non-desiccating ponds varied in depth through the survey period (reference smooth, *p* < 0.001; figure 3*b*). The two pond types varied in their seasonal patterns in depth (difference smooth, *p* < 0.001), with a more dramatic seasonal variability in desiccating ponds over non-desiccating ponds (figure 3*b*).

Host and parasite dynamics were highly variable across the circannual cycle (August 2021–July 2022), with differing patterns between desiccating and non-desiccating ponds. *Bulinus* abundance number closely mirrored water depth seasonality (figure 3*c*), with nonlinear patterns of abundance over time in non-desiccating ponds (reference smooth, *p* = 0.003) and a more dramatic boom and bust pattern in desiccating ponds over non-desiccating ponds (differential smooth, *p* < 0.001). While desiccating waterbodies had a potentially elevated transmission risk due to rapidly growing snail population numbers following the onset of rain, non-desiccating waterbodies had a subdued but more constant snail abundance.

**Figure 4.**
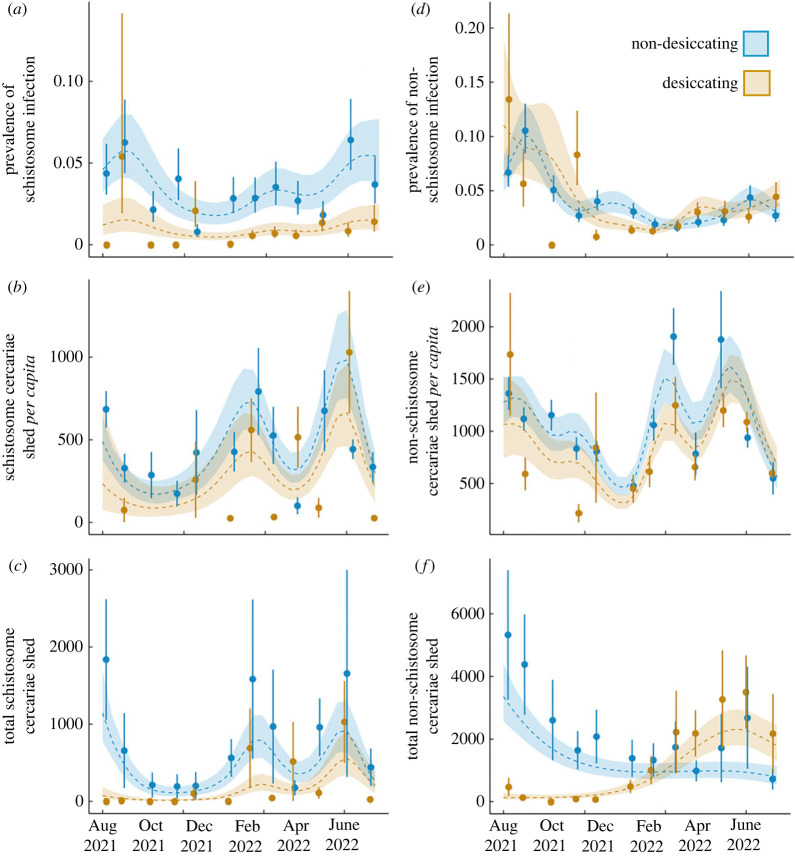
GAMMs representing circannual (*a*) schistosome prevalence, (*b*) schistosome per capita cercariae observed, (*c*) schistosome total cercaria release per survey, (*d*) non-schistosome prevalence, (*e*) non-schistosome *per capita* cercariae observed, and (*f*) non-schistosome total cercaria observed per survey in desiccating and non-desiccating ponds. The shaded region surrounding the line represents the best fit ± 1 s.e. Points represent monthly means and standard error. All patterns were significantly nonlinear and different between the two ephemerality categories, except per capita cercariae observed was equivalent between two pond types.

Schistosome infection patterns (figure 4*a*) indicate a nonlinear fluctuation in non-desiccating ponds in terms of prevalence (reference smooth, *p* < 0.001), and consistent, significantly lower peaks in non-desiccating ponds after from October 2021 (differential smooth, *p* = 0.044). A similar nonlinear pattern is seen of *per capita* (figure 4*b*) and total (figure 4*c*) schistosome cercariae observed in non-desiccating ponds (reference smooth, *p* < 0.001). However, while total cercarial release has significantly lower peaks in desiccating ponds (differential smooth, *p* = 0.014; figure 4*c*), *per capita* release is equivalent across both pond types (differential smooth, *p* = 0.444; figure 4*b*). In both pond types, there are two primary prevalence and cercariae peaks following the onset of rain (peak rainfall in mid-January 2022); one in mid-rainy season and another early in the dry season. Yearly risk of transmission of schistosomes is substantially lower in desiccating ponds than non-desiccating ponds, with snails being 4.6 times more likely to be infected in the latter (binomial GLM; *p* < 0.001).

The non-schistosome trematode GAMMs indicate significantly nonlinear seasonal patterns of infection prevalence and cercarial release in non-desiccating ponds (reference smooths, *p* < 0.001; figure 4*d–f*). Non-desiccating ponds had an early-mid rainy season peak (December 2021–January 2022) of infection prevalence whereas the rainy season infection peak of desiccating ponds is later in March–May 2022 (differential smooth, *p* < 0.001; figure 4*d*). In non-desiccating ponds, *per capita* cercarial release varies over time (reference smooth, *p* < 0.001), and does not differ in seasonality from desiccating ponds (differential smooth, *p* = 0.121; figure 4*e*). In addition, there is a large peak of total cercarial release only in desiccating ponds, from January 2022 preceding the next dry season (differential smooth, *p* < 0.001; figure 4*f*). *Bulinus* snails are 1.6 times more likely to be infected by non-schistosome trematodes in non-desiccating ponds than desiccating ponds (binomial GLM; *p* < 0.001), which is a substantially smaller difference than schistosomes.

**Figure 5.**
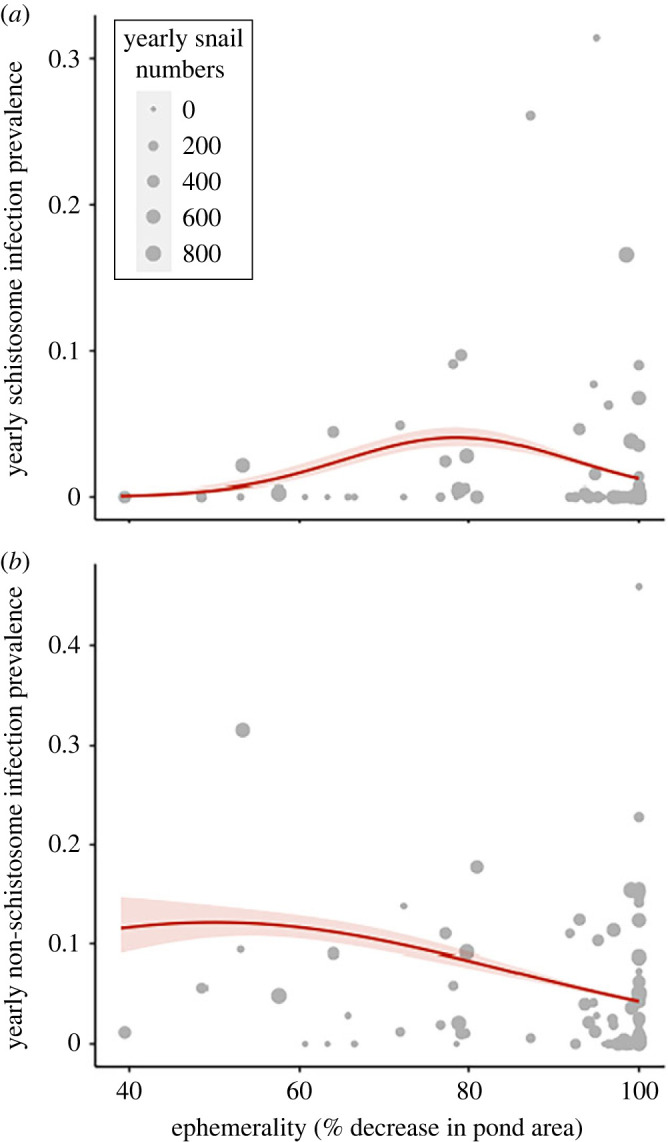
Overall yearly (*a*) schistosome and (*b*) non-schistosome trematode infection prevalence in across an ephemerality gradient (% decrease in pond area in the dry season). The shaded region surrounding the line represents the best fit with 95% CI. Points represent yearly prevalence in each pond and the size is indicative of the number of snails collected in each pond per year. Schistosome prevalence peaks at intermediate ephemerality (approx. 80% area decrease, binomial GLM, *p* < 0.01). Non-schistosome prevalence peaks at low ephemerality (approx. 50% area decrease, binomial GLM, *p* < 0.01).

Cumulative yearly prevalence varied considerably with intensity of pond ephemerality (% reduction in pond area in the dry season) for both parasite groups. Yearly schistosome infection had a significantly nonlinear relationship with ephemerality, where prevalence peaked in ponds at intermediate ephemerality (maximum at approx. 80% reduction in area, binomial GLM; *p* < 0.001; figure 5*a*). Yearly non-schistosome infection prevalence peaked in ponds with lower ephemerality (approx. 50% reduction in area) and decreased steadily with increasing ephemerality (binomial GLM; *p* < 0.001; figure 5*b*).

A small number of snails infected with either schistosomes (*n* = 5) or non-schistosome (*n* = 15) trematodes were identified less than 60 days following a survey where the pond was dry (table 1). This provides evidence that snails do aestivate while infected, albeit rarely, and emerge and shed parasites. Infected aestivators are likely to have also emerged from non-desiccating ponds at higher rates contributing to the first schistosome infection peak following the rainy season (figure 4*a*).

**Table 1.** Case studies of infections identified less than 60 days after ponds were dry. Information is provided on parasite type, pond location, number of concurrent dry surveys of ponds prior to infections, number of snails in survey with infections, number of infected snails in survey with infections and number of days before infections were detected since pond was last identified as dry.

parasite type	pond name, village, district	number of dry surveys	number of snails in survey	number of infected snails in survey	number of days since last dry survey
schistosome	Lambo la Mhana, Ngudama, Misungwi	1	100	1	31 (1 survey prior)
	Lambo la Nzego Matunge, Shilalo, Misungwi	3	14	1	54 (2 surveys prior)
	Lambo la Joseph Malyengete, Isole, Sengerema	3	10	3	54 (2 surveys prior)
non-schistosome	Kwa Fumu, Nyang'holongo, Misungwi	1	3	1	21 (1 survey prior)
	Lambo la Sospeter Walwa, Ikungumhulu, Misungwi	1	32	13	32 (1 survey prior)
	Lambo la Nzego Matunge, Shilalo, Misungwi	3	3	1	32 (1 survey prior)

## 4. Discussion

All sites in a seasonally desiccating landscape are not equal and this leads to a spectrum of dormancy conditions for their occupying populations. The prevalence of both schistosome and non-schistosome parasites was dampened in ponds that desiccated for at least one month of the year when compared to ponds that held standing water year-round. In the case of schistosomes, infection prevalence peaked in ponds with intermediate ephemerality, while non-schistosome prevalence decreased with increasing ephemerality. In addition, parasite infection prevalence and cercarial release was not constant through the year, resulting in multiple ‘hot moments' of transmission risk for hosts. Schistosome transmission risk peaks twice following the onset of rain synchronously across both pond types, regardless of ephemerality. The first could be associated with infections persisting through aestivation and second could be a result of new infections following the onset of rain. Seasonal transmission patterns of non-schistosome parasites, on the other hand, are highly asynchronous across the two ephemerality categories. While *per capita* cercariae observed of either parasite group did not differ between the two pond categories, there was seasonal variability with several peaks of per capita and total cercarial observed which would be likely to lead to higher parasite exposure risk for hosts entering ponds. As East Africa will continue to experience more extreme dry season conditions with global change, we could expect spatial and temporal shifts in transmission risk with increases in pond ephemerality.

Similar to previous studies [7,28], we found that pond desiccation was not a deterrent to *Bulinus* snail populations which have an impressive capacity for population rebounding in desiccating ponds. This could be the result of factors such as escalated feeding behaviour and reproduction of snails emerging from aestivation [38] and their populations being regulated by a more diverse community of competitors and predators in non-desiccating ponds than could be supported in the harsh habitat seasonality of desiccating ponds [24]. While the potential for recovery of intermediate host snails following aestivation is clear, their aestivation ecology and impact on infection are still largely understudied, especially in the field [21]. Aestivation imposes physiological constraints on snails [39] and infection enhances this physiological stress [40], which likely explains the lower infection rates in desiccating than non-desiccating waterbodies. In addition, selection may favour snails resistant to infection and thus more likely to survive aestivation. We did recover snails infected with schistosomes or non-schistosome trematodes that emerged from aestivation alive at low rates in desiccating ponds. And we only documented surviving infected aestivators in ponds that had shorter desiccation periods (i.e. one to three months), suggesting that prolonged desiccation decreases the likelihood of survival of infected snails. However, the timeframe of our study (beginning in the dry season) prohibited our capacity to accurately evaluate if the total duration of pond desiccation impacted host and parasite outcomes. Additionally, we were unable to identify infected snails emerging from aestivation in non-desiccating ponds, where we may expect at higher numbers than in desiccating ponds. Identifying habitat factors that promote and deter aestivation success of snails, whether infected or not, remains a line of inquiry for the future.

Another possible determinant of low infection rates in desiccating ponds is definitive host use. Shorter hydroperiods (length of time a waterbody has standing water) limit host exposure to and contamination of the water, disrupting the transmission cycle. Even if snails were to get infected, they have a limited amount of time to develop patent infections and thereafter, survival is limited if snails undergo aestivation with infections [40]. It is, thus, surprising that ponds with the longest hydroperiods and biggest area, such as dams, had low schistosome transmission risk. These larger ponds are often designated for cattle use in large densities, for activities such as cattle washing stations, and thus may be less suitable for human use. We are unable to confirm this definitively as we did not genetically differentiate *S. haematobium*, *S. bovis* and their hybrids. Future studies would benefit from identifying specific schistosome parasite species and evaluating their variable prevalence with different pond restrictions and host use. In contrast to schistosomes, we do see peak risk for non-schistosome parasites in large ponds. Non-schistosome parasites are in general at higher infection prevalence and intensities across space and time in our study. Cattle interact with ponds at higher densities and are far more likely to urinate/defecate in and around ponds than humans, at any time of year and regardless of the water depth. In addition, anthelmintics are often variably effective with increasing parasitic infection and timing of infection and we might expect cattle to have higher exposure and intensity of infections due to their indiscriminate water use and contamination, and densities.

This still leaves an open question as to why ponds with intermediate ephemerality favour schistosome transmission. These ponds have longer hydroperiods than desiccating ponds, which perhaps creates periods with concentrated exposure/contamination risk as water depths lower in the dry season. Alternatively, snails have been observed to aestivate in non-desiccating ponds as standing water level decreases, likely triggered by unfavourable water conditions [21]. However, the presence of standing water may create gentler aestivation conditions in the soil, limiting mortality of infected snails when compared to desiccating ponds. Experimental approaches and field observations may help elucidate the mechanisms underlying elevated parasite success in these intermediate pond types.

One important caveat of our study is that snail abundance and infection is not always a directly correlated with human schistosome infection rates as snail infection rates are typically very low [41] and infections also depend on behavioural and immunological factors. While schistosome studies will always be aided by assessments of human infection rates, our snail and cercarial release metrics provide an estimate of exposure rates experienced by hosts entering a waterbody. Additionally, we saw distinct seasonal peaks in transmission risk of schistosomes and non-schistosomes. However, as our study was conducted over a single year with a slightly atypical rainfall pattern, the long-term generality of these patterns must be interpreted with caution. This highlights the importance of long-term studies for the repeatability of infection peaks and troughs, and an understanding of their underlying predictors, especially with global change.

Global change is impacting the timing and intensity of dormancy periods with crucial impacts on populations during active periods of the year's cycle. For example, Penczykowski *et al*. [11] demonstrated that overwintering dormancy conditions have become less harsh with increasing winter temperatures, resulting in higher plant-fungi prevalence in the springs that follow. East Africa is expected to experience increased desertification with climate change [29], with variable possible outcomes on human schistosome geographical distribution [42] and transmission potential [43] at large spatial scales. At a more local scale, Mutuku *et al*. [44] demonstrated that a decade-long drought resulted in a near elimination of *S. haematobium* in a coastal Kenyan village, suggesting that the transition from moderate to extreme ephemerality could interrupt transmission cycles. With increasing drought risk, ponds are likely to experience longer dormancy periods and shorter periods with standing water. In our study, peak schistosome transmission risk was seen at intermediate intensities of ephemerality which could result in highly variable future outcomes depending on the pond desiccation patterns; they could have higher or lower transmission risk depending on their natural tendency for drying. In the case of non-schistosome parasites, we found increasing ephemerality was correlated with decreasing cumulative infection risk suggesting that transmission may not be well sustained in an increasingly desiccating landscape. Thus, we might expect to see an interruption of the transmission cycle of human and other waterborne parasites resulting in potentially beneficial outcomes for human, livestock and wildlife diseases. However, it is hard to predict how all parties of these disease transmission cycles will respond to the desiccation of their landscape. Short generation times in trematodes and snails may result in adaptation to longer dormancy and shorter active periods, such as hardier dormancy phenotypes and faster reproductive cycles. Species may also experience shifts in geographical ranges in response to a changing climate, for example another human schistosome species (*S. mansoni*) has been detected at higher elevations than previously recorded in Uganda [45].

Humans have shown a history of largely small-scale mitigation to a desiccating landscape, with variable responses by different stakeholders and at different scales [30]. The creation and periodic modification of these ponds, for example, was for the purpose of improving year round water availability due to a history of droughts [46], as well as to provide water for an increasingly irrigated agricultural sector in Sub-Saharan Africa [47]. Thus, further droughts could result in the creation of more such ponds or enlarging of existing ones to increase year-round water supplies. Either outcome has the potential to provide habitat that favours schistosome and other trematode infection risk. Alternatively, with increasing human populations there may not be sufficient space, and this may provide an opportunity for alternative water storage and conservation methods, which may also be beneficial for human public and environmental health. Some methods used include terracing, rainwater harvest tanks, sub-surface storage and afforestation [30]. In the meantime, lessons from this natural laboratory of varying ephemerality could be used to mitigate infection risk: ponds drying out completely reduces the transmission risk of schistosome infection. Seasonal pumping of water from these types of ponds to above ground water sources to dry out the soil may intensify aestivation conditions like in desiccating ponds and limit parasite survival. Additional disturbance of soil conditions, such as sun-drying, ploughing and tilling, may further limit survival capacity [27,48]. Any such interventions should only be made with conscientious dialogue with village members as to not disrupt their water practices and availability in the dry season.

Highly ephemeral waterbodies have the potential to disrupt the transmission cycle of human and other animal trematodes. Factors such as pond size, depth, shape, substrate, nutrient availability, vegetation density and seasonal use by definitive hosts interact with a rapidly changing climate to determine infection outcomes. The dissimilar pattern of transmission risk between these two parasite groups across space and time documented here asserts the necessity of taking a One Health approach to identifying specific mechanisms underlying their infection success in a desiccating landscape.

## Data Availability

The data associated with this publication (along with the associated R code) are available on Dryad Digital Repository: https://doi.org/10.5061/dryad.mpg4f4r4t [49].
